# B cell polygenic risk scores associate with anti-dsDNA antibodies and nephritis in systemic lupus erythematosus

**DOI:** 10.1136/lupus-2023-000926

**Published:** 2023-10-16

**Authors:** Anna Hedenstedt, Sarah Reid, Ahmed Sayadi, Maija-Leena Eloranta, Elisabeth Skoglund, Karin Bolin, Martina Frodlund, Karoline Lerang, Andreas Jönsen, Solbritt Rantapää-Dahlqvist, Anders A Bengtsson, Anna Rudin, Øyvind Molberg, Christopher Sjöwall, Johanna K Sandling, Dag Leonard

**Affiliations:** 1 Rheumatology, Department of Medical Sciences, Uppsala University, Uppsala, Sweden; 2 Department of Biomedical and Clinical Sciences, Division of Inflammation and Infection/Rheumatology, Linköping University, Linkoping, Sweden; 3 Department of Rheumatology, Oslo University Hospital, Oslo, Norway; 4 Rheumatology, Department of Clinical Sciences, Lund University, Lund, Sweden; 5 Department of Public Health and Clinical Medicine/Rheumatology, Umeå University, Umea, Sweden; 6 Department of Rheumatology and Inflammation Research, University of Gothenburg Sahlgrenska Academy, Gothenburg, Sweden

**Keywords:** B cells, Lupus Erythematosus, Systemic, Autoantibodies, Polymorphism, Genetic, Lupus Nephritis

## Abstract

**Objective:**

B cell function and autoantibodies are important in SLE pathogenesis. In this work, we aimed to investigate the impact of cumulative SLE B cell genetics on SLE subphenotype and autoantibody profile.

**Methods:**

Female patients with SLE (n=1248) and healthy controls (n=400) were genotyped using Illumina’s Global Screening Array. Two polygenic risk scores (PRSs), one representing B cell genes and the other B cell activation genes, were calculated for each individual using risk loci for SLE in genes assigned to B cell-related pathways according to the Kyoto Encyclopedia of Genes and Genomes, Gene Ontology and Reactome Databases.

**Results:**

Double-stranded DNA (dsDNA) antibodies were more prevalent among patients with a high compared with a low SLE B cell PRS (OR 1.47 (1.07 to 2.01), p=0.018), and effect sizes were augmented in patients with human leucocyte antigen (HLA) risk haplotypes HLA-DRB1*03:01 and HLA-DRB1*15:01 (DRB1*03/15 −/− (OR 0.99 (0.56 to 1.77), p=0.98; DRB1*03/15 +/− or −/+ (OR 1.64 (1.06 to 2.54), p=0.028; and DRB1*03/15 +/+ (OR 4.47 (1.21 to 16.47), p=0.024). Further, a high compared with a low B cell PRS was associated with low complement levels in DRB1*03/15 +/+ patients (OR 3.92 (1.22 to 12.64), p=0.022). The prevalence of lupus nephritis (LN) was higher in patients with a B cell activation PRS above the third quartile compared with patients below (OR 1.32 (1.00 to 1.74), p=0.048).

**Conclusions:**

High genetic burden related to B cell function is associated with dsDNA antibody development and LN. Assessing B cell PRSs may be important in order to determine immunological pathways influencing SLE and to predict clinical phenotype.

**WHAT IS ALREADY KNOWN ON THIS TOPIC**

- B cell abnormalities are important contributors in SLE and lupus nephritis pathogenesis.
- Genetic profiling through polygenic risk scores has been shown useful to stratify patients with SLE according to dominating molecular disease mechanism, but has not been investigated for specific disease manifestations.

**WHAT THIS STUDY ADDS**

- Here, we demonstrate that high B cell polygenic risk scores are associated with development of anti-double-stranded DNA antibodies, low complement and lupus nephritis.

**HOW THIS STUDY MIGHT AFFECT RESEARCH, PRACTICE OR POLICY**

- Our results suggest a method to identify patients with a B cell-dominated disease, which could be important in prediction of organ damage and choice of therapy.

## Introduction

SLE is an inflammatory multisystem disorder that affects approximately 3–5 per 100 000 person-years.^1^ SLE pathogenesis is characterised by production of antibodies directed at nuclear antigens, formation of immune complexes and increased activity in the type I interferon system.^2 3^ This results in damage to multiple organ systems and tissues, and gives rise to a broad range of clinical manifestations. One of the most severe is lupus nephritis (LN), which affects 40–50% of patients with SLE^4^ and leads to end-stage renal disease in up to 11% of cases.^5^

While it is widely accepted that SLE develops in genetically predisposed individuals exposed to triggering environmental factors, the genetic background is complex and in most cases, specific genes cannot alone explain disease development in a patient.^6^ Until today, approximately 180 SLE susceptibility loci have been identified at genome-wide significance (5×10^−8^).^7^

Genome-wide association study (GWAS) data enable construction of a polygenic risk score (PRS), which analyses the weighted effect of disease-related single nucleotide polymorphisms (SNPs) in each individual in order to quantify their genetic burden.^8^ Our group has previously shown that patients with SLE with a high PRS have an earlier disease onset, an increased risk of early and more severe organ damage and impaired survival.^8^

We have also demonstrated that pathway-specific PRSs can be adopted to further stratify patients according to dominating molecular disease mechanism.^9^ In the latter work, high B cell and T cell signalling PRSs were associated with development of organ damage according to the Systemic Lupus International Collaborating Clinics (SLICC) Damage Index.^10^

B cells are known to be involved in SLE pathogenesis by various mechanisms leading to loss of self-tolerance and production of autoreactive antibodies,^11 12^ and several therapeutic agents aimed at B cells have already been implicated in treatment of SLE and LN.^13 14^

Elevated titres of antibodies against double-stranded DNA (dsDNA) have previously been linked to higher disease activity in LN and with overall organ damage, and rising levels of anti-dsDNA antibodies have been shown to accurately predict severe lupus flares within 6 months.^15 16^ Anti-dsDNA antibodies and other antibodies bind self-antigens and form immune complexes that are deposited in organs and tissues. Deposition in kidneys results in complement activation, immune cell infiltration and inflammation, which leads to destruction of the kidney parenchyma.^17^

Several genetic risk factors have been highlighted in context of LN susceptibility.^18^ Important associations include genetic variations in *BANK1* and several SNPs that affect *STAT4*.^19 20^ Moreover, polymorphisms in the human leucocyte antigen (HLA) genes, particularly the HLA-DRB1 region, have been strongly linked to SLE in general, and specifically to LN for some haplotypes.^21^ HLA-DRB1 variants have also recently been associated with distinct subgroups of SLE with diverse clinical presentation and autoantibody profile.^22^

In the current study, we aimed to investigate associations between SLE PRSs with genes limited to B cell function, autoantibody production, complement levels and organ manifestations, as well as the significance of high PRS in patients with HLA risk variants HLA-DRB1*03:01 and HLA-DRB1*15:01.

## Methods

### Patients and healthy controls

A total of 1248 female patients with SLE recruited from six rheumatology clinics in Sweden and Norway were included. All patients were of European descent and fulfilled at least one of the 1982 American College of Rheumatology (ACR-82), ACR-97 or the 2012 SLICC classification criteria (SLICC-12) sets.^23–25^ The control group included 400 female blood donors from Sweden, matched for ethnicity and age.^26^

Clinical data were collected from patients’ medical records, including information on disease duration, ACR classification criteria, low complement according to the SLICC-12 criteria,^24^ autoantibody ever positivity and renal disorder according to ACR-82.^23^ Kidney biopsy was classified according to the WHO^27^ or International Society of Nephrology/Renal Pathology Society (ISN/RPS) 2003^28^ classification systems, and the one with the most severe histopathological pattern was included. Patient characteristics are summarised in table 1.

**Table 1:** Patient characteristics (n=1248)

Female	1248 (100)
Age at diagnosis*	35 (3–82)
Age at follow-up*	53 (18–92)
Deceased at follow-up	183 (11.1)
ACR criteria^23^	
ACR 1: malar rash	711 (56.7)
ACR 2: discoid rash	276 (22.0)
ACR 3: photosensitivity	865 (69.2)
ACR 4: oral ulcer	350 (28.0)
ACR 5: arthritis	971 (77.4)
ACR 6: serositis	471 (37.5)
ACR 7: renal disorder	373 (29.7)
ACR 8: neurological disorder	117 (9.3)
ACR 9: haematological disorder	794 (64.2)
ACR 10: immunological disorder	860 (69.5)
ACR 11: ANA	1233 (98.3)
Antibodies	
dsDNA	618 (62.6)
Sm	116 (11.5)
Cardiolipin†	382 (32.5)
Lupus anticoagulant	232 (27.8)
β2-glycoprotein I†	211 (25.1)
SSA	227 (34.6)
SSB	268 (21.3)
Complement	
Low C3/C4/CH50‡	540 (55.4)

Data are number (%).

All patients were of European descent and fulfilled any of the ACR-82, ACR-97 or SLICC-12 classification criteria sets.

*Median (min–max).

†IgG or IgM.

‡Low complement levels defined according to the SLICC-12 criteria.^24^

ACR, American College of Rheumatology; dsDNA, double-stranded DNA; SLICC, Systemic Lupus International Collaborating Clinics; SSA, Sjögren’s syndrome-related antigen A; SSB, Sjögren’s syndrome-related antigen B.

### Genetic information and construction of PRSs

Patients and controls were genotyped using Illumina’s Global Screening Array. Quality control (QC) was performed to exclude low-quality samples and variants. Samples with a call rate below 95% were excluded. The HapMap project data were included in a principal component analysis, which was used to remove samples more than 5 SD from the European populations. To identify genetic relatedness between samples, PLINK was used to estimate the pairwise identity by descent (IBD).^29^ Samples with an IBD higher than 0.1875 were removed. Samples with heterozygosity rates more than 5 SD from the mean were excluded. For each sample, the inbreeding coefficient F was estimated; if F was lower than 0.8 and the sample was annotated as a male or if F was higher than 0.2 and the sample was annotated a female, it was excluded. After QC, a total of 1248 female patients with SLE were kept for downstream analysis. Variants with a call rate below 98%, with a minor allele frequency below 0.01 or which failed the Hardy-Weinberg equilibrium test (p<1e−4), were all filtered out. After QC, a total of 503 123 single nucleotide variants were preserved. Data were imputed using the Haplotype Reference Consortium V.r1.1 reference dataset and the ‘pre-phase with EAGLE2 and impute’ pipeline at the Sanger imputation server.^30^ After imputation, 5 222 989 variants were obtained.

A total of 181 SLE-associated SNPs at GWAS significance (p<5×10^−8^) from studies including >1000 patients and patients of European ancestry were identified through the GWAS catalogue^31^ (accessed 1 January 2022) and published articles (table 2). The SNP with the highest OR for SLE was selected for each risk locus. Following exclusion of SNPs in linkage disequilibrium (LD) (r^2^>0.2), 133 of these SNPs were identified in our dataset. Next, SNPs were assigned to genes using functional mapping and annotation of genetic associations,^32^ and genes were assigned to pathways based on their functions according to the Kyoto Encyclopedia of Genes and Genomes,^33^ Gene Ontology^34 35^ and Reactome Databases.^36^ SNPs assigned to genes related to B cell function were included in two PRSs as described below. In addition, two general SLE PRSs were constructed, one including all non-HLA SLE SNPs (n=115) and one excluding the 20 SNPs included in the B cell PRSs (n=95). The PRSs were calculated by multiplying the number of risk alleles present in each individual with the natural logarithm of OR for SLE from published articles at each risk locus. When the minor allele was protective for SLE, the major allele was defined as the risk allele and counted in the PRSs, ensuring that the directionality for SLE risk was consistently positive. The sum of all products was defined as each individual’s PRS.

**Table 2:** SNPs included in the PRSs

SNP	Risk allele	Gene	RAF*	OR†	SLE B cell PRS	SLE B cell activation PRS	Publication‡
rs2230926	G	*TNFAIP3*	0.06	2.03	×	×	Morris *et al*^44^
rs11073328	T	*RASGRP1*	0.13	1.94	×		Armstrong *et al*^45^
rs8076347	T	*IKZF3*	0.05	1.93	×	×	Lessard *et al*^46^
rs150518861	A	*LAT2*	0.02	1.66	×	×	Julià *et al*^47^
rs7726414	T	*SKP1*	0.05	1.45	×		Bentham *et al*^48^
rs4810485	T	*CD40*	0.26	1.43	×	×	Langefeld *et al*^49^
rs13277113	A	*BLK*	0.32	1.39	×		Lee *et al*^50^
rs6679677	A	*PTPN22*	0.15	1.39	×		Bentham *et al*^48^
rs7829816	T	*LYN*	0.16	1.30	×	×	Harley *et al*^51^
rs2286672	T	*PSMB6*	0.08	1.25	×		Bentham *et al*^48^
rs9899849	A	*GPS2*	0.26	1.21	×	×	Wang *et al*^52^
rs930297	A	*GRB2*	0.09	1.20	×		Langefeld *et al*^49^
rs10028805	G	*BANK1*	0.28	1.20	×	×	Bentham *et al*^48^
rs3024505	A	*IL10*	0.18	1.19	×	×	Gateva *et al*^53^
rs597808	A	*PTPN11*	0.51	1.18	×		Bentham *et al*^48^
rs1432296	T	*REL*	0.19	1.18	×		Langefeld *et al*^49^
rs907715	G	*IL21*	0.31	1.16	×	×	Hughes *et al*^54^
rs1966115	A	*IL7*	0.27	1.14	×	×	Langefeld *et al*^49^
rs6871748	T	*IL7R*	0.28	1.12	×	×	Wang *et al*^52^
rs3087243	G	*CTLA4*	0.38	1.12	×	×	Wang *et al*^52^

*RAF in the patient cohort.

†ORs for SLE from published articles used in calculation of the PRSs.

‡Articles from which SNPs were identified had >1000 patients in study and reported SNPs were at GWAS significance level (p<5×10^–8^).

GWAS, genome-wide association study; PRSs, polygenic risk scores; RAF, risk allele frequency; SNPs, single nucleotide polymorphisms.

HLA variants HLA-DRB1*03:01 and HLA-DRB1*15:01 were assessed in patients using tag SNPs rs1269852 and rs3135388, respectively.^8 37 38^

### Statistical analysis

PRSs were analysed both as continuous variables, and by grouping PRSs into high and low, defining high as PRSs in the fourth quartile and low as quartiles 1–3, respectively. Logistic regression was used to assess differences in prevalence rates between groups, and Student’s t-test was employed to compare mean PRSs between patients and controls. Cox regression was used to assess survival until LN onset in patients with and without dsDNA antibodies. Years of disease duration was included as a covariate in all logistic regression analyses and Cox regression was adjusted for age at disease onset. A 95% CI was used, and p values below 0.05 were considered significant. All analyses were performed using statistical software SPSS (V.28.0.1.0).

## Results

### PRS and SLE prevalence

A total of 20 genes associated with B cell function were included in the construction of two PRSs: one covering all 20 B cell-related genes (SLE B cell PRS) and one covering 12 genes with functions limited to B cell activation (SLE B cell activation PRS). Genes and SNPs included in the PRSs are summarised in table 2.

**Figure 1:**
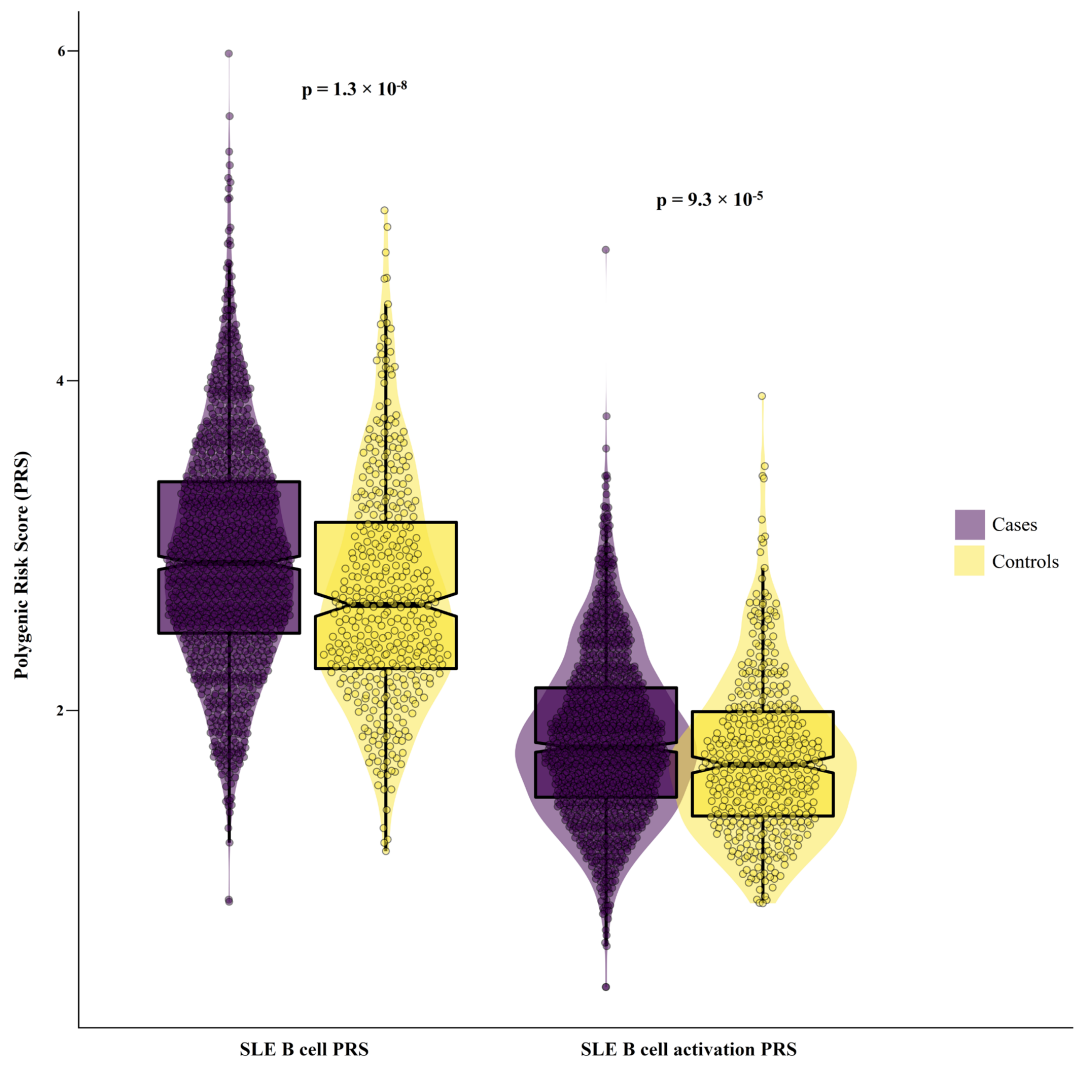
SLE B cell PRS and SLE B cell activation PRS in female patients and controls. PRS, polygenic risk score.

The two PRSs followed a Gaussian distribution (online supplemental figure 1) in both patients and controls, and the mean SLE B cell PRS was significantly higher in patients 2.92 (2.88 to 2.96) than controls 2.69 (2.62 to 2.76), p=1.26×10^−8^. Similar but smaller differences were seen in the mean B cell activation PRS 1.81 (1.78 to 1.83) and 1.70 (1.65 to 1.74) for patients and controls, respectively (p=9.28×10^−5^) (figure 1). SLE was more prevalent in individuals with a high compared with low SLE B cell PRS (OR 1.64 (1.38 to 1.95), p=2.0×10^−8^), and the same was found for the SLE B cell activation PRS (OR 1.58 (1.25 to 2.01), p=1.3×10^−4^).

10.1136/lupus-2023-000926.supp1 Supplementary data

### PRS and autoantibody associations

To examine PRS performance and associations between PRS and clinical manifestations, we analysed associations between the B cell PRS and prevalence of all ACR-82 criteria and selected autoantibodies. We found a high SLE B cell PRS to be associated with both higher prevalence of immunological disorder (OR 1.44 (1.08 to 1.93), p=0.014) and anti-dsDNA antibodies (OR 1.47 (1.07 to 2.01), p=0.018) (table 3). The prevalence of all ACR criteria and antibodies investigated is summarised in table 1. In order to compare the effect of the B cell PRS on anti-dsDNA antibody development with the overall genetic risk for SLE, we calculated a general SLE PRS including 115 non-HLA SNPs and a similar PRS excluding the 20 B cell SNPs. Results show lower nominal OR for the SLE PRS excluding the B cell SNPs than for the general SLE PRS (OR 1.39 (1.02 to 1.90), p=0.035 and OR 1.60 (1.17 to 2.19), p=0.0034, respectively).

**Table 3:** Associations between clinical manifestations and SLE B cell PRS (n=1248)

	OR* (95% CI)	P value
ACR criteria^23^		
ACR 1: malar rash	0.97 (0.74 to 1.26)	0.81
ACR 2: discoid rash	0.77 (0.56 to 1.07)	0.12
ACR 3: photosensitivity	0.93 (0.70 to 1.22)	0.59
ACR 4: oral ulcer	0.94 (0.71 to 1.26)	0.68
ACR 5: arthritis	0.99 (0.73 to 1.34)	0.95
ACR 6: serositis	1.19 (0.91 to 1.56)	0.21
ACR 7: renal disorder	1.20 (0.91 to 1.59)	0.20
ACR 8: neurological disorder	1.16 (0.75 to 1.80)	0.50
ACR 9: haematological disorder	1.05 (0.80 to 1.37)	0.74
ACR 10: immunological disorder	**1.44 (1.08 to 1.93)**	**0.014**
ACR 11: ANA	1.04 (0.38 to 2.87)	0.94
Antibodies		
dsDNA	**1.47 (1.07 to 2.01)**	**0.018**
Sm	0.91 (0.57 to 1.46)	0.71
Cardiolipin*†	1.01 (0.76 to 1.35)	0.93
Lupus anticoagulant	1.08 (0.76 to 1.53)	0.68
β2-glycoprotein†	1.20 (0.84 to 1.72)	0.33
SSA	1.17 (0.89 to 1.53)	0.26
SSB	1.09 (0.80 to 1.50)	0.58
Complement		
Low C3/C4/CH50‡	1.21 (0.90 to 1.64)	0.21

Values in bold indicate p<0.05.

*ORs for PRSs in the highest quartile compared with quartiles 1–3.

†IgM or IgG.

‡Low complement levels defined according to the SLICC-12 criteria.^24^

ACR, American College of Rheumatology; dsDNA, double-stranded DNA; PRS, polygenic risk score; SLICC, Systemic Lupus International Collaborating Clinics; SSA, Sjögren’s syndrome-related antigen A; SSB, Sjögren’s syndrome-related antigen B.

Next, we hypothesised that the associations between high SLE B cell PRS, immunological disorder and dsDNA antibodies would differ in patients with HLA risk variants. Therefore, we stratified patients in three subgroups according to HLA haplotype and compared the prevalence of immunological disorder and dsDNA antibodies in the high versus low B cell PRS groups. The subgroups included: patients without HLA-DRB1*03:01 and HLA-DRB1*15:01 (DRB1*03/15 −/−) (n=354), those positive for the HLA-DRB1*03:01 or the HLA-DRB1*15:01 tag SNPs in one allele (DRB1*03/15 +/− or −/+) (n=656) and those positive for both HLA-DRB1*03:01 and HLA-DRB1*15:01 (DRB1*03/15 +/+) (n=143).

**Figure 2:**
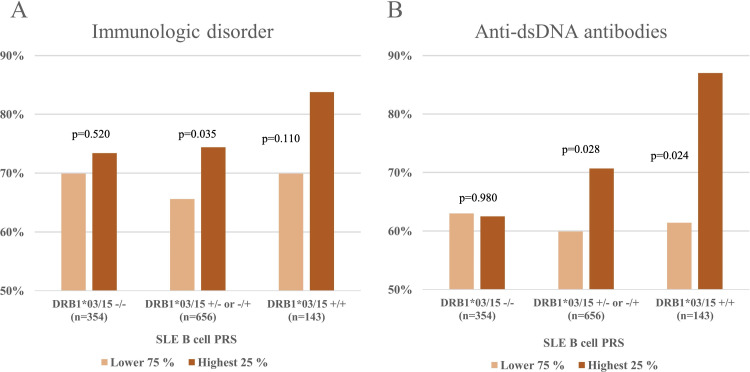
Associations with SLE B cell PRS, immunological disorder (ACR-82) and anti-dsDNA antibodies in HLA haplotype subgroups. Female patients with SLE were stratified into three groups according to HLA type (HLA-DRB1*03:01 and HLA-DRB1*15:01 +/− or −/+, HLA-DRB1*03:01 and HLA-DRB1*15:01 +/+, and HLA-DRB1*03:01 and HLA-DRB1*15:01 −/−). Each group was then divided into two groups based on the patients’ SLE B cell PRSs (highest quartile or quartiles 1–3). Prevalence of immunological disorder according to the ACR-82 criteria (A) and prevalence of dsDNA antibodies (B) was then calculated. Tag SNP rs1269852 was used as a proxy for HLA-DRB1*03 and tag SNP rs3135388 for HLA-DRB1*15. ACR, American College of Rheumatology; dsDNA, double-stranded DNA; HLA, human leucocyte antigen; PRS, polygenic risk score; SNP, single nucleotide polymorphism.

Similar to what was observed in the entire patient cohort, we found that for patients with DRB1*03/15 +/− or −/+, prevalence of both immunological disorder and dsDNA antibodies was higher in those with a high compared with a low SLE B cell PRS (OR 1.54 (1.03 to 2.29), p=0.035 and OR 1.64 (1.06 to 2.54), p=0.028, for immunological disorder and dsDNA antibodies, respectively) (figure 2). In the same analysis, we also observed greater effect sizes for patients with DRB1*03/15 +/− or −/+, compared with patients negative for both HLA risk variants (online supplemental file 1). The largest effect size was observed for anti-dsDNA antibodies in patients positive for both HLA-DRB1*03:01 and HLA-DRB1*15:01 (OR 4.47 (1.21 to 16.47), p=0.024).

For patients with HLA-DRB1*03/15 +/+, we also found that prevalence of low complement levels, defined according to SLICC-12 criteria,^24^ was higher in patients with a high SLE B cell PRS (83%), compared with patients with low PRS (56%) (OR 3.92 (1.22 to 12.64), p=0.022) (online supplemental table 1).

### PRS and LN associations

Given the association between a high SLE B cell PRS and anti-dsDNA antibodies, and the known association between anti-dsDNA antibodies and LN,^17^ we hypothesised that our B cell PRSs would predict higher prevalence of LN.

In our cohort, a total of 370 (29.7%) patients had LN according to the ACR-82 criteria. LN was more prevalent among patients with dsDNA antibodies (36%) than those without (17%) (OR 2.80 (2.03 to 3.85), p=8.0×10^−11^). Also, LN onset occurred in average 6 years earlier in patients with dsDNA antibodies, compared with patients without (HR 2.89 (1.77 to 4.72), p=2.1×10^−5^).

A kidney biopsy was performed in 62% of patients with LN and the most common histopathological pattern observed was diffuse proliferative nephritis (class IV) (47%), followed by focal proliferative nephritis (class III) (21%). While dsDNA antibody prevalence was numerically higher in all groups with biopsy-verified LN compared with no LN (ACR-82), the only significant association between dsDNA antibodies and LN was found for patients with class III–IV (OR 4.66 (2.78 to 7.80), p=5.2×10^−9^).

For patients with a high SLE B cell PRS, prevalence of LN according to the ACR-82 criteria was numerically higher compared with patients with a low PRS, but the difference did not reach statistical significance (OR 1.20 (0.91 to 1.59), p=0.20). However, the prevalence of renal disorder according to the ACR-82 definition was significantly higher in patients with a high compared with a low B cell activation PRS (OR 1.32 (1.00 to 1.74), p=0.048) (online supplemental table 2). Furthermore, we observed a close to significant association between high SLE B cell activation PRS and higher prevalence of class III–IV nephritis (OR 1.39 (0.96 to 2.00), p=0.079). Associations between high SLE B cell PRS or SLE B cell activation PRS and other histopathological patterns could not be demonstrated. All investigated associations between clinical manifestations, autoantibodies and SLE B cell activation PRS are summarised in online supplemental table 2.

## Discussion

Our study demonstrates associations between high genetic burden in B cell-related pathways, autoantibody production and LN in SLE, providing clues on a potential tool to assess future risk of organ damage of patients with SLE.

Important findings include first, increased prevalence of SLE in individuals with high B cell-related genetic risk. It has previously been shown that the weighted effect of multiple SLE-related SNPs likely plays a more important role in SLE pathogenesis than any individual high-risk genes alone.^8^ It is therefore not surprising to find a smaller subset of SLE risk loci to have a similar effect on disease risk. However, the limitation to genes in B cell-related pathways adds important information on what pathways might be dominating in pathogenesis for some patients.

Second, it was shown that patients with SLE with a high SLE B cell PRS were more likely to fulfil the immunological disorder criterion and to present with anti-dsDNA antibodies. This supports the hypothesis that generation of autoreactive antibodies is promoted by genetic aberrations in B cells to a significant extent. A variety of B cell abnormalities have already been described in SLE. Notably, it has been demonstrated that patients with SLE display imbalance in B cell populations in peripheral blood, altered function in immunosuppressive regulatory B cells and expansion in subsets of autoreactive effector B cells.^11 12^ Our findings are consistent with previous knowledge, and suggest a method to identify patients with the highest degree of B cell-related genetic burden, and therefore the highest risk of developing autoantibody-related organ damage.

No significant association was observed between the B cell PRS and other autoantibodies, but a trend, with ORs above 1, was shown for anti-Sjögren’s syndrome-related antigen A (SSA), anti-Sjögren’s syndrome-related antigen B (SSB) and anti-β2-glycoprotein I antibodies. This result could be related to low power in the analysis, but could also be due to a specific effect of the PRS on anti-dsDNA antibody production. Compared with SSA and SSB antibodies, titres of anti-dsDNA antibodies vary over time. One could therefore speculate that gene variants included in the PRS affect B cells and plasma cells, increasing production of anti-dsDNA antibodies above the level of detection. However, further studies are needed to clarify if this is the case.

When comparing the effect of the B cell PRS with the overall non-HLA genetic risk for SLE on anti-dsDNA antibody development, we found the OR to be nominally higher for the SLE PRS. However, when removing the B cell SNPs from this PRS, the nominal OR was lower than that of the B cell PRS. This in spite of the B cell PRS including a considerably smaller number of SNPs (20 vs 95). These results suggest that the average B cell-associated SLE locus may contribute more to development of anti-dsDNA antibodies than the average SLE risk SNP in general. But also, as can be expected, that other SLE risk loci are important for the production of autoantibodies.

Third, HLA risk variants HLA-DRB1*03:01 and HLA-DRB1*15:01 were shown to augment the effect of a high SLE B cell PRS in anti-dsDNA antibody development. The underlying mechanism of HLA haplotype in context of SLE susceptibility is yet unknown. However, our results indicate that high genetic burden related to B cells plays a more central role for patients with certain HLA types, perhaps because the combination of increased B cell reactivity and HLA-mediated antigen presentation predisposes for dsDNA antibody production, more than either risk factor alone.

Here, we also demonstrated that patients with HLA-DRB1*03/15 +/+ and high SLE B cell PRS had higher prevalence of low complement levels. Reduction in C3, C4 and CH50 levels is well described in SLE, and has earlier been inversely associated with rising levels of dsDNA antibodies.^39 40^ Our results could be a reflection of a subgroup of patients’ genetic predisposition to produce anti-dsDNA antibodies, subsequent formation of immune complexes and complement consumption.

Although a significant association between high SLE B cell PRS and LN could not be shown here, we demonstrate a significantly higher prevalence of LN in patients with high PRSs related to B cell activation. One explanation to this might be the relatively larger contribution of the *BANK1* SNP rs10028805 in the SLE B cell activation PRS, compared with the SLE B cell PRS. We have previously shown variants in *BANK1* to be associated with LN in a cohort partially overlapping with the present study population.^20^ The B cell activation PRS was not as strongly associated with nephritis as presence of dsDNA antibodies. This might not be surprising given the well-known strong association between these antibodies and LN.^15^ Further studies are needed to clarify the role of the B cell activation PRS, available early in disease, in nephritis prediction.

Strengths of this study include a large cohort and extensive clinical information with well-defined SLE classification criteria. Another advantage is the long-time follow-up, which however has allowed for the use of different methods and cut-off levels for measurement of autoantibodies. Limiting analyses to women allowed for analysis of a cohort with as much genetic similarities as possible. However, the limitation to female gender and European ancestry was also a weakness, as the results are only applicable to this group. The clinical phenotype of SLE varies in populations of different descent, trending towards a more active disease with more organ damage and higher mortality in patients of African ancestry, compared with patients of white populations.^41^ The same is known for males with SLE, who are known to more often develop LN and to have a generally more aggressive disease.^42^

With increasing knowledge of genetic and immunological pathways influencing LN pathogenesis, it is likely that the future of LN management lies in precision medicine. An important step forward is the identification of biomarkers or instruments enabling early recognition of patients genetically predisposed to develop LN, which could motivate closer monitoring or even pre-emptive therapy.^43^ Assessing B cell PRSs could be one important component in developing such instruments. Future areas of research would be to verify the results presented here in an independent cohort. Further, it will be important to examine if and how the B cell PRSs influence gene expression in cell subsets such as B cells and cells in kidney tissue. It would also be interesting to investigate if it could be used to predict clinical response to B cell targeting therapy in SLE. To conclude, assessing B cell polygenic risk may be important in order to determine immunological pathways influencing SLE. However, further studies are needed to clarify the relationship between genetics and clinical phenotype in SLE, and to improve PRS performance.

## Data Availability

Data are available upon reasonable request. The datasets generated during the current study are not publicly available due to them containing information that could compromise research participants’ privacy and consent, but are available from the corresponding authors on reasonable request and on a collaborative basis.
