# Supplementary material for: B cell polygenic risk scores associate with anti-dsDNA antibodies and nephritis in systemic lupus erythematosus
Source: Lupus Sci Med. 2023 Oct 16;10(2):e000926. doi: 10.1136/lupus-2023-000926 (PMC10582984; doi:10.1136/lupus-2023-000926)
Supplement: Supplementary data [file lupus-2023-000926supp001.pdf]

Supplementary tables and figures

Supplementary table 1. SLE B cell PRS in HLA subgroups

	n	%	OR (95 % CI)*	p
**HLA-DRB1*03:01 and HLA-DRB1*15:01 negative patients (DRB1*03/15 -/-) (n=354)**				
Immunologic disorder (ACR-82)	246	70.5	1.20 (0.69-2.11)	0.52
dsDNA antibodies	176	62.9	0.99 (0.56-1.77)	0.98
Low C3/C4/CH50**	148	54.0	1.07 (0.61-1.88)	0.823
**HLA-DRB1*03:01 or HLA-DRB1*15:01 positive patients (DRB1*03/15 +/- or -/+) (n=656)**				
Immunologic disorder (ACR-82)	439	67.9	**1.54 (1.03-2.29)**	**0.035**
dsDNA antibodies	323	62.5	**1.64 (1.06-2.54)**	**0.028**
Low C3/C4/CH50**	277	54.4	1.14 (0.76-1.72)	0.529
**HLA-DRB1*03:01 and HLA-DRB1*15:01 positive patients (DRB1*03/15 +/+) (n=143)**				
Immunologic disorder (ACR-82)	103	73.6	2.24 (0.85-5.91)	0.11
dsDNA antibodies	71	67.0	**4.47 (1.21-16.47)**	**0.024**
Low C3/C4/CH50**	66	61.7	**3.92 (1.22-12.64)**	**0.022**

Values in bold indicate $p < 0.05$. *ORs for SLE B cell PRS in the highest quartile compared to quartile 1-3. **Low complement levels according to the SLICC classification criteria.[1] SLE, systemic lupus erythematosus; PRS, polygenic risk score; HLA, Human leukocyte antigen; ACR, American College of Rheumatology; dsDNA, double-stranded DNA; SLICC, Systemic Lupus International Collaborating Clinics.

Supplementary table 2. Associations between clinical manifestations and SLE B cell activation PRS (n=1248)

	OR (95 % CI)	p
**ACR criteria[2]**		
ACR 1: Malar rash	1.26 (0.97-1.65)	0.87
ACR 2: Discoid rash	0.98 (0.72-1.34)	0.89
ACR 3: Photosensitivity	1.02 (0.78-1.36)	0.86
ACR 4: Oral ulcer	1.04 (0.79-1.39)	0.76
ACR 5: Arthritis	1.82 (0.61-1.10)	0.18
ACR 6: Serositis	1.20 (0.92-1.57)	0.18
ACR 7: Renal disorder	**1.32 (1.00-1.74)**	**0.048**
ACR 8: Neurologic disorder	0.94 (0.60-1.49)	0.80
ACR 9: Hematologic disorder	1.08 (0.83-1.42)	0.56
ACR 10: Immunologic disorder	1.12 (0.84-1.49)	0.44
ACR 11: ANA	1.81 (0.31-2.11)	0.66
**Antibodies**		
dsDNA	1.10 (0.82-1.50)	0.51
Sm	0.94 (0.60-1.49)	0.80
Cardiolipin**	1.06 (0.80-1.40)	0.71
Lupus anticoagulant	0.97 (0.69-1.38)	0.87
β2-glycoprotein**	1.13 (0.79-1.63)	0.50
SSA	1.09 (0.83-1.42)	0.55
SSB	1.04 (0.76-1.42)	0.82
**Complement**		
Low C3/C4/CH50***	1.33 (0.99-1.79)	0.060

Values in bold indicate $p < 0.05$. *ORs for PRSs in the highest quartile compared to quartile 1-3. **IgM or IgG. ***Low complement levels according to the SLICC classification criteria.[1] SLE, systemic lupus erythematosus; PRS, polygenic risk score, ACR, American College of Rheumatology; ANA, anti-nuclear antibodies, SSA, Sjögren's-syndrome-related antigen A; SSB, Sjögren's-syndrome-related antigen B; SLICC, Systemic Lupus International Collaborating Clinics.

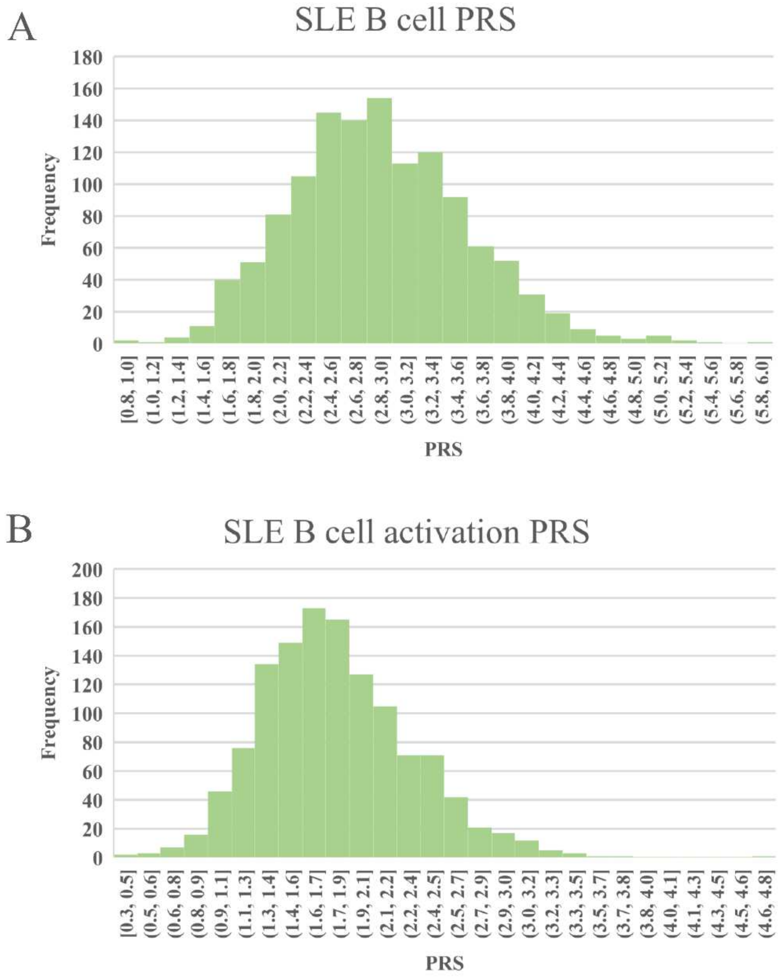

Supplementary figure 1. Distribution of PRSs calculated for 1248 female SLE patients. The SLE B cell PRS (A) included 20 genes related to B cell function and the SLE B cell activation PRS (B) included a subset of 12 of these genes. SLE, systemic lupus erythematosus; PRS, polygenic risk score.

References:

1. Petri M, Orbai AM, Alarcon GS, *et al.* Derivation and validation of the Systemic Lupus International Collaborating Clinics classification criteria for systemic lupus erythematosus. *Arthritis Rheum* 2012;64:2677-2686.
2. Tan EM, Cohen AS, Fries JF, *et al.* The 1982 revised criteria for the classification of systemic lupus erythematosus. *Arthritis Rheum* 1982;25:1271-1277.
